# Factors associated with home blood pressure monitoring practices among patients with hypertension in Putrajaya, Malaysia: A cross-sectional study

**DOI:** 10.51866/oa.889

**Published:** 2025-09-11

**Authors:** Luqman Melissha, Siew Mooi Ching, Mohamad Fadzilah, Hanum Mohd Yusof Hazira

**Affiliations:** 1 MD, MMed (Fam Med), PhD, Klinik Kesihatan Presint 18, Presint 18, Putrajaya, Wilayah, Persekutuan Putrajaya, Malaysia.; 2 Department of Family Medicine, Faculty of Medicine and Health Sciences, Universiti Putra Malaysia, Serdang, Selangor, Malaysia.; 3 Malaysian Research Institute on Ageing, Faculty of Medicine and Health Sciences, Universiti Putra Malaysia, Serdang, Selangor, Malaysia.; 4 Department of Medical Sciences, School of Healthcare and Medical Sciences, Sunway University, Bandar Sunway, Petaling Jaya, Selangor, Malaysia.; 5 Research Centre of Excellence, Nutrition and Non-communicable Diseases, Faculty of Medicine and Health Sciences, Universiti Putra Malaysia, Serdang, Selangor, Malaysia. Email: sm_ching@upm.edu.my; 6 MD, MMed (Fam Med), Klinik Kesihatan Presint 18, Presint 18, Putrajaya, Wilayah Persekutuan Putrajaya, Malaysia.; 7 Department of Family Medicine, Faculty of Medicine and Health Sciences, Universiti Putra Malaysia, Serdang, Selangor, Malaysia.; 8 MBBCh BAO, MMed (Fam Med), Department of Family Medicine, Faculty of Medicine and Health Sciences,Universiti Putra Malaysia, Serdang, Selangor, Malaysia.; 9 MD, MMed (Fam Med), Klinik Kesihatan Presint 18, Presint 18, Putrajaya, Wilayah Persekutuan Putrajaya, Malaysia.

**Keywords:** Home blood pressure monitoring, Hypertension, Practice, Factors, Malaysia

## Abstract

**Introduction:**

This study primarily aimed to determine the factors associated with home blood pressure monitoring (HBPM) practices among patients with hypertension in Putrajaya, Malaysia.

**Methods:**

A cross-sectional study was conducted among patients with hypertension aged 18 years and above in a government health clinic in Putrajaya. An adopted and validated questionnaire on the knowledge, attitude and practice of HBPM was utilised. A total of 338 patients with hypertension were recruited.

**Results:**

The median age of the participants was 52 years. Approximately 95.3%, 87.3%, 61.2%, 54.4% and 49.4% were Malay, were married, completed tertiary education, were men and were government servants, respectively. The prevalence of good HBPM knowledge, good attitude towards HBPM and HBPM practice among the participants was 79.0%, 57.1% and 81.7%, respectively. Having medical insurance (adjusted odds ratio [aOR]: 3.05, 95% confidence interval (CI): 1.47— 6.29, P=0.003), having diabetes mellitus (DM) (aOR: 4.58, 95% CI: 2.47-8.49, P<0.01), staying with household members (aOR: 2.95, 95% CI: 1.07-8.10, P=0.036), being a non-smoker (aOR: 3.72, 95% CI: 1.41-9.80, P=0.008) and engaging in physical activity (aOR: 2.47, 95% CI: 1.314.65, P=0.005) showed significant associations with HBPM practice.

**Conclusion:**

The knowledge and practice of HBPM among patients with hypertension in Putrajaya, Malaysia, were excellent. The significant associated factors of HBPM practice were having medical insurance, having DM, staying with household members, being a non-smoker and being physically active. This study should be extended to the whole nation to obtain a true reflection of HBPM practice among Malaysians.

## Introduction

Hypertension is a prevalent non-communicable disease (NCD) and a leading cause of premature death worldwide. It is a significant risk factor for cardiovascular disease (CVD)^1^ and chronic kidney disease (CKD),^2^ with the prevalence of each increasing with age. Hypertension affects two-thirds of the 1.28 billion population mainly from low- and middle-income countries (LMICs)^3^ and is the most prevalent lifelong illness seen in primary healthcare, with approximately one in every eight people receiving anti-hypertensive medication.^4^ The prevalence of hypertension has been on the rise, particularly in LMICs, while high-income countries have been experiencing a modest decrease due to effective management and preventive measures.^1^ These discrepancies in the patterns of hypertension prevalence imply that healthcare systems in LMICs might be facing a rapidly expanding burden of hypertension and its related complications.^5^

In Malaysia, the prevalence of hypertension among adults aged 18 years and above was 32.7% in 2011^6^ and decreased only minimally over the years, having been 30.3% in 2015,^7^ 30.0% in 2019^8^ and 29.2% in 2023.^9^ Despite the declining trend, the prevalence of hypertension in Malaysia remains greater than that in other Southeast Asian nations, such as Singapore (23.5%), Thailand (24.7%), Indonesia (26.5%) and the Philippines (28.0%).^10^ In contrast to the national trend, Putrajaya, Malaysia’s federal administrative capital, showed an increasing trend, with hypertension prevalence rising from 24.1% in 2015^7^ and 24.7% in 2019% to 24.9% in 2023.^9^

The regional increase in the hypertension burden indicates the need for an effective measure to control and manage hypertension.

Home blood pressure monitoring (HBPM) is the measurement of blood pressure (BP) outside a clinical environment^11^ and has emerged as a practical and cost-effective approach to manage hypertension.^12^ It has the ability to enhance BP control, requiring less doctor visits, influence treatment decisions and improve prognosis.^13^ Additionally, HBPM can aid in the identification of the white-coat effect and masked hypertension.^14^ Globally, the rate of HBPM usage ranges from 30.0% to 70.0%, influenced by factors such as socioeconomic factors (educational level, occupation, marital status, higher household income, medical insurance and cohabitation), clinical characteristics, mental health status, recommendations from healthcare professionals, healthcare accessibility and cultural attitudes towards self-monitoring.^15-22^ The worldwide prevalence of HBPM is estimated to range from 19.1% to 74.7%.^16-20^, ^22-24^

To date, data on HBPM practice and its associated factors among patients with hypertension in the Malaysian setting are limited, particularly in Putrajaya. Therefore, the main objective of this study was to determine the factors associated with HBPM practice among patients with hypertension in Putrajaya, Malaysia. Identifying the determinants of HBPM practice can help provide valuable insights into policies and interventions aimed at improving hypertension management in Malaysia.

## Methods

### Study setting

This cross-sectional study was conducted from November 2023 to May 2024 among patients with hypertension at a government health facility in Putrajaya. The selection of Putrajaya as the study location was based on its greater median family income relative to Malaysia25 and the rising prevalence of hypertension,7-9 which correlates with an increased ownership of HBPM devices.

### Study population

Malaysian individuals over 18 years of age, diagnosed with hypertension by a doctor for a duration exceeding 6 months, attending follow-up appointments at the selected health clinic and prescribed anti-hypertensive medication were included in the study. Pregnant or breastfeeding individuals, patients undergoing haemodialysis, patients taking over-the-counter or traditional drugs, patients with psychiatric disorders that disrupt social and daily functioning and individuals unable to read Malay or English were excluded.

### Sample size

The sample size was calculated using G*Power version 3.1 based on study findings that 87.2% of patients with hypertension owned HBPM devices and that 73.4% of them were practising HBPM.^24^ Based on an effect size of 0.5 (twosided), a power of 80.0% and a significance level of 0.05, the minimum required sample size calculated was 278. Given an 80.0% response rate, the final sample size required for this study was 334.

### Study instrument

The questionnaire utilised Google Forms and was administered with assistance. It comprised seven sequential sections: 1) sociodemographic background, 2) hypertension background, 3) medical history, 4) medical treatment background, 5) lifestyle, 6) BP and anthropometric measurements and 7) knowledge of, attitudes towards and practice of HBPM (Appendix 1), adapted from different studies.^16,23,26^ The knowledge component comprised 16 questions, with a minimum score of 0 and a maximum score of 16. Respondents who achieved 13 marks or more (at least 80.0% of the total mark of 16) were considered as having good knowledge, as recommended in most studies.^27^

For the attitude component, a 5-point Likert scale, ranging from *totally disagree* to *totally agree,* with a total of nine questions, was used. It had a minimum score of 9 and a maximum score of 45. A score of 5 was given when respondents answered *strongly agree* with positive-statement items and a score of 1 when they answered *strongly disagree*. The scoring was vice versa for negative-statement items. Respondents who obtained a mark of 36 or more (at least 80.0% of the total mark of 16) were considered as having good attitudes.^28^

The practice component comprised 16 questions assessing HBPM practices. Nevertheless, no evaluation was conducted for this section, as we intended to examine the characteristics of practising HBPM descriptively. BP and weight were measured using automated BP measuring devices and digital weighing scales by OMRON. These devices were pre-tested and complied with the quality control standard.

The standard operating procedures for BP and weight measurement were based on the global guidelines.^29, 30^

The questionnaire was translated into Bahasa Malaysia, and content validation was conducted among six experts including three family medicine specialists, an internal medicine physician, a public health medicine specialist and a dietician. The overall content validity index was 0.94.

### Data collection

Systematic random sampling was applied to recruit respondents from a pool of patients with hypertension who attended the hypertension clinic at the Putrajaya Pres int 18 Health Clinic during the data collection period. The sampling fraction (K) for the sample size was obtained by dividing the estimated number of patients attending the hypertension clinic into the number of samples. Since the sample size (n) was 334, drawn from a population (N) of 1000 patients (calculated as 200 patients per week over 5 weeks), a sampling interval (K) of approximately 3 (1000/334) was calculated.

The first individual was selected using the lottery method, generated using Microsoft Excel, and the rest were selected at a regular interval (every third patient) using systematic random sampling. If the first person rejected to participate in the study, the next third person was selected. The process was continued throughout the data collection period until the targeted number of respondents was achieved. Data were automatically generated in Google Excel upon the completion of the self-administered online questionnaire.

### Data analysis

Data were entered into IBM SPSS Statistics for Windows, version 26 (IBM Corp., Armonk, N.Y., USA). Categorical data were reported as frequencies and percentages. Conversely, continuous data were tested for normality of their distribution. Normally distributed continuous data were presented as means and standard deviations (SDs), while non-normally distributed data were presented as medians and interquartile ranges (IQRs). Multiple logistic regression (MLR) was used to identify the determinants of HBPM practice after adjusting for confounding factors using the forward stepwise (conditional) method.

Several assumptions were required for the logistic regression analysis: a dichotomous dependent variable, no duplication of data, clear separation of outcomes between sample groups, absence of multicollinearity as assessed by tolerance and variance inflation factor values and achievement of at least 80.0% of the calculated sample size. The independent variables with a P-value of <0.25 from the single logistic regression were included in the MLR to identify the determinants of the dependent variables. The level of significance was set at a P-value of <0.05.

## Results

A total of 338 respondents were recruited into the study. Table 1 shows the sociodemographic and clinical characteristics of the respondents. The median age was 51.96 years (IQR: 10.89), with men constituting 54.4% of the study population. The median monthly income was RM 6246.89 (IQR: 5031.15). The majority of the respondents were married (87.3%), were Malay (95.3%) and stayed with family members (93.5%). About three-quarters received tertiary education (61.2%), and almost half were government servants (49.4%). Only 34.0% of the respondents had medical insurance coverage.

About 68.9% of the respondents had diabetes mellitus (DM), and 82.0% had dyslipidaemia. The majority of the respondents did not have stroke, CVD or CKD. The median duration of hypertension was 6.33 years (IQR: 5.96), and 86.4% had a family history of hypertension. About half of the respondents took one type of anti-hypertensive medication (55.6%). Approximately 96.7% were never admitted to the hospital for the past 6 months, and 95.3% were advised by their doctor to practise HBPM.

All respondents had good mental health (100.0%); 87.9% were non-smokers; 99.4% abstained from alcohol; and 51.2% performed regular physical activity. The median BMI was 30.0 kg/m2 (IQR: 5.81). The mean ± SD BP at the clinic was 139/86±14/10 mmHg, while the median BP at home was 131/83 mmHg (IQR: 9/7 mmHg). About 79.0% had a good HBPM knowledge; 57.1% had a good attitude towards HBPM; and 81.7% practised HBPM.

**Table 1:** Sociodemographic and clinical characteristics of the respondents (N=338).

Variable		n	%	Median	IQR
Age (year)				51.96	10.89
Monthly income (RM)				6246.89	5031.15
Sex	Male	184	54.4		
	Female	154	45.6		
	Single	30	8.9		
Marital status	Married	295	87.3		
	Divorced	13	3.8		
	Malay	322	95.3		
Race	Chinese	4	1.2		
	India	7	2.1		
	Other	5	1.5		
Companion status	Alone	22	6.5		
	Staying with others	316	93.5		
	No formal education	4	1.2		
Educational level	Primary education	12	3.6		
	Secondary education	115	34.0		
	Tertiary education	207	61.2		
	Unemployed	62	18.3		
	Retiree	55	16.3		
Occupation	Self-employed	22	6.5		
	Private sector employee	32	9.5		
	Government servant	167	49.4		
Medical insurance		115	34.0		
Diabetes mellitus		233	68.9		
Dyslipidaemia		278	82.2		
Stroke		9	2.7		
CVD		7	2.1		
CKD		7	2.1		
Duration of hypertension	<1 year	42	12.4		
	≥1 year	296	87.6		
Duration of hypertension (year)				6.33	5.96
Family history of hypertension		292	86.4		
Number of anti-hypertensive agents	1	188	55.6		
	2	118	34.9		
	≥3	32	9.5		
Admission for the past 6 months		11	3.3		
Recommendation by a doctor to practise HBPM		322	95.3		
Good mental health		338	100.0		
Non-smoker		297	87.9		
Non-alcohol consumer		336	99.4		
Physical activity	Less active	165	48.8		
	Moderately or highly active	173	51.2		
Body mass index (kg/m^2^)				30.36	5.81
SBP reading at the clinic (mmHg)				138.95^†^	14.01^ℇ^
DBP reading at the clinic (mmHg)				86.36^†^	10.20^ℇ^
SBP reading at home (mmHg)				131.40	9.44
DBP reading at home (mmHg)				83.01	7.05
Good knowledge^*^ of HBPM		267	79.0		
Good attitude# towards HBPM		193	57.1		
HBPM practice		275	81.7		

CVD: cardiovascular disease, CKD: chronic kidney disease, HBPM: home blood pressure monitoring, IQR: interquartile range

† Mean value

ℇ Standard deviation

* Good knowledge is defined as a mark of 13 or more (at least 80.0% of the total score of 16) in the knowledge component of the questionnaire.

# Good attitude is defined as a mark of 36 or more (at least 80.0% of the total score of 45) in the attitude component of the questionnaire.

About 68.9% of the respondents had diabetes mellitus (DM), and 82.0% had dyslipidaemia. The majority of the respondents did not have stroke, CVD or CKD. The median duration of hypertension was 6.33 years (IQR: 5.96), and 86.4% had a family history of hypertension. About half of the respondents took one type of anti-hypertensive medication (55.6%). Approximately 96.7% were never admitted to the hospital for the past 6 months, and 95.3% were advised by their doctor to practise HBPM.

All respondents had good mental health (100.0%); 87.9% were non-smokers; 99.4% abstained from alcohol; and 51.2% performed regular physical activity. The median BMI was 30.0 kg/m^2^ (IQR: 5.81). The mean ± SD BP at the clinic was 139/86±14/10 mmHg, while the median BP at home was 131/83 mmHg (IQR: 9/7 mmHg). About 79.0% had a good HBPM knowledge; 57.1% had a good attitude towards HBPM; and 81.7% practised HBPM.

Table 2 shows the characteristics of HBPM practice among 275 patients with hypertension. All respondents utilised automated BP devices; 80.0% calibrated their HBPM devices; and 54.9% checked for any leakage of the cuff. Approximately 81.8% of the respondents measured their BP with a frequency of four times or more in a month, and 78.9% had their recent BP measurement within 7 days. About 90.2% were able to recall the most recent BP reading. Consistent pre-measurement resting practices were noted among 97.1% of the respondents. About 76.0% measured BP in the morning after waking, while 49.5% measured BP in the evening.

**Table 2:** General characteristics of the respondents who practised HBPM (n=275).

Variable	n	%
Used automated HBPM devices	275	100.0
Had calibrated their HBPM devices	220	80.0
Had checked for any leakage of the cuff	151	54.9
Frequency of BP measurements in a month		
<4 times	50	18.2
≥4 times	225	81.8
Most recent BP measurement		
≥7 days ago	58	21.1
<7 days ago	217	78.9
Had recalled the most recent BP reading	248	90.2
Had rested 30 minutes before the BP measurement	267	97.1
Had practised BP measurement after waking up in the morning	209	76.0
Had practised BP measurement in the evening	136	49.5
Had practised to record the BP measurement	216	78.5
Had recorded the BP reading in a logbook	166	60.4
Had recorded the BP reading on their phone or computer	79	28.7
Had informed the BP reading to their doctor during follow-up	266	96.7
Had sought treatment when the BP reading was too high	246	89.5

Among the total participants, 78.5% recorded their BP readings, with 60.4% using a logbook and 28.7% charting on their phone or computer. Nearly all (96.7%) reported their readings to their physicians during follow-ups, and 89.5% sought treatment when readings exceeded 180/110 mmHg. The majority of the respondents preferred to record their BP readings in a logbook (60.4%) rather than on their phone or computer (28.7%). Almost all respondents also informed the doctor of their BP readings during follow-up (96.7%), and the majority sought treatment when their BP reading was too high at more than 180/110 mmHg (89.5%).

Table 3 shows the significant factors associated with the practice of HBPM evaluated using single and MLR. The patients with hypertension with medical insurance had 3.0 times higher odds of practising HBPM as compared to those without medical insurance (adjusted odds ratio [aOR]: 3.05, 95.0% CI: 1.47-6.29, P=0.003). The respondents who stayed with household members had 3.0 times higher odds of practising HBPM than those who stayed alone (aOR: 2.95, 95.0% CI: 1.07-8.10, P=0.036). The respondents with DM were 4.6 times more likely to practise HBPM as compared to those without DM (aOR: 4.58, 95.0% CI: 2.47-8.49, P<0.001). The non-smokers and passive smokers had 3.7 times higher odds of practising HBPM than the smokers (aOR: 3.72, 95.0% CI: 1.41-9.80, P=0.008). The respondents who did moderate-to-highly vigorous physical activity were 2.5 times more likely to practise HBPM than the sedentary respondents (aOR: 2.47, 95.0% CI: 1.31-4.65, P=0.005).

**Table 3:** Logistic regression analysis of the factors affecting the practice of HBPM.

Variable	Crude OR	95.0% CI	P-value	aOR	95.0% CI	P-value
Medical insurance status						
Yes	3.05	1.47, 6.33	**0.003** ^*^	3.05	1.47, 6.29	**0.003** ^*^
No	1.00			1.00		
Companion status						
Staying with others	2.89	1.04, 8.01	**0.042** ^*^	2.95	1.07, 8.10	**0.036** ^*^
Staying alone	1.00			1.00		
Diabetes mellitus status						
Yes	4.73	2.51, 8.90	**<0.001** ^*^	4.58	2.47, 8.49	**<0.001** ^*^
No	1.00			1.00		
Smoking status						
Non-smoker or passive smoker	3.54	1.32, 9.51	**0.012** ^*^	3.72	1.41, 9.80	**0.008** ^*^
Smoker	1.00			1.00		
Physical activity status						
Moderately to highly active	2.38	1.26, 4.50	**0.008** ^*^	2.47	1.31, 4.65	**0.005** ^*^
Less active	1.00			1.00		

OR: odds ratio, aOR: adjusted odds ratio, CI: confidence interval

* Significant at P<0.05

## Discussion

This study found a high prevalence of good HBPM knowledge (79.0%), indicating the effectiveness of the current healthcare programme in recommending HBPM as a selfmonitoring tool in hypertension management. This prevalence is higher than that in previous studies conducted in Nigeria (54.7%),^31^ Saudi Arabia (41.0%)^32^ and Ethiopia (31.5%).^16^

However, the prevalence of positive attitudes towards HBPM was only 57.1%, indicating potential gaps in patient education. The findings of other studies are inconsistent. For instance, a cross-sectional study performed in Ethiopia demonstrated that 18.8% of respondents agreed that self-monitoring BP is important; 17.0% agreed that self-monitoring BP could benefit them; 10.5% agreed that self-monitoring BP is not accurate; and 22.5% agreed to recommend self-monitoring BP to other patients with hypertension.^16^ Another cross-sectional study conducted in Nigeria found that 88.0% of respondents agreed that self-monitoring of BP is important.^31^

The prevalence of HBPM practice among the patients with hypertension in this study was 81.7%, higher than that reported in other countries such as Italy (74.7%),^17^ Poland (64.0%),^18^ Greece (53.0%),^24^ the United States (51.2%),^22^ Canada (47.1%),^20^ Ethiopia (31.5%),^16^ Singapore (24.0%)^23^ and the Czech Republic (19.1%).^19^ The high prevalence of HBPM practice in this study could be due to the patients attending the specialised hypertension clinics, in which activity oriented on spreading knowledge about HBPM benefits is provided by the treating doctor.^19^ In this study, 95.3% of the respondents were recommended by their doctor to practise HBPM, which is also reported in a previous study.^22^

The other reasons for the high prevalence of practising HBPM could be due to the popularity of HBPM in urban areas such as Putrajaya and the availability of self-monitoring of BP in the community, especially at local pharmacies and grocery stores.^33^ Another reason could be the use of digital BP measurement devices, which are more user-friendly, save time and have good reliability of BP measurement as compared to old and manual mercury sphygmomanometer BP measurement devices.^17^

In this study, all respondents (100.0%) used electronic BP devices at home; the same proportion was reported in Singapore (100.0%),^23^ Poland (100.0%)^18^ and the United States (100.0%),^22^ but the proportion is larger than that in Greece (33.6%)^24^ and Italy (58.0%).^17^ The majority of the respondents calibrated their devices (80.0%) (almost the same proportion as that in a study conducted in Singapore [83.3%]),^23^ measured their BP four times or more in a month (81.8%) (almost the same as that in a study conducted in Ethiopia [90.7%]),^16^ took their last BP measurement less than 7 days ago (78.9%), recalled the most recent BP reading (90.2%), took their BP measurement after waking up in the morning (76.0%), rested before the BP measurement (97.1%), recorded the BP measurement (78.5%) (larger than the proportion recorded in a study conducted in Ethiopia [27.1%]),^16^ informed the doctor about their BP reading during follow-up (96.7%) (larger than the proportion recorded in a study performed in the United States [50.0%]),^22^ sought treatment when their BP reading was too high (89.5%) and recorded their BP reading in a logbook (60.4%) instead of a phone or computer.

Previous studies have demonstrated different types of HBPM practice items. Among these studies, the most information about the practice of HBPM was found in the study conducted by Tan et al. among patients with hypertension in polyclinics in Singapore.^23^ Due to this incomplete information of HBPM practice in the previous literature, this study provides comprehensive information on HBPM practice among patients with hypertension for better reference.

Several factors were identified as significant determinants of HBPM among the patients with hypertension. The respondents who had medical insurance had 3.1 times higher odds of practising HBPM (95% CI: 1.47-6.29). This finding is similar to the OR of 3.56 (95% CI: 1.39-10.53) from a study conducted in Ethiopia,^16^ emphasising the role of financial security in enabling HBPM practice.^34^ The respondents who stayed with household members were 3.0 times more likely to practise HBPM (95% CI: 1.07-8.10). This finding is supported by a study conducted in Canada, with an aOR of 2.00 (95% CI: 1.41-2.80),^20^ highlighting the role of family support in chronic disease management particularly in terms of medication compliance, diet control and disease monitoring (i.e. glucose level and BP).^35,36^

The odds of practising HBPM among the respondents with DM were 4.6 times higher than those among the respondents without DM (95% CI: 2.47-8.49). This finding is consistent with previous reports from the United States (aOR: 1.71, 95% CI: 0.93-3.15),^33^ Singapore (aOR: 1.44, 95% CI: 0.69-3.11),^23^ Poland (aOR: 1.72, 95% CI: 1.48-1.98)^18^ and Ethiopia (aOR: 3.93, 95% CI: 1.35-10.32).^16^ This may be because individuals with co-morbidities are more likely to adopt proactive health behaviours, including HBPM, after receiving recommendations from healthcare providers.^37^

Lifestyle factors, such as non-smoking (aOR: 3.72, 95% CI: 1.41-9.80, P=0.008) and physical activity (aOR: 2.47, 95% CI: 1.31-4.65, P=0.005), were also significant determinants of HBPM. These findings are consistent with those of another study conducted in Poland (aOR: 0.64 for smoker respondents, 95% CI: 0.57-0.72; aOR: 1.44 for physically active respondents, 95% CI: 1.27—1.65).^18^ Non-smoking individuals have good health literacy and are more aware of their health, which prompt them to do everything to make sure that they are healthy.^38^ Physically active individuals tend to practise a healthy lifestyle to ensure good control of their BP^39^

### Study limitations

To date, this is the first study to report the determinants of HBPM among patients with hypertension. The main limitation of the study is the narrow spectrum of socioeconomic populations represented among the respondents, with the majority being of the Malay race in Putrajaya. This homogeneity may limit the generalisability of the study findings to the broader Malaysian population, as the results could differ significantly if other ethnic groups were included. Including a more diverse sample would provide a more comprehensive understanding of HBPM practices across different socioeconomic and ethnic groups in Malaysia.

Other limitations include the non-exploration of the respondents who did not practise HBPM. In the questionnaire, we did not provide an item to assess the reason for not practising HBPM, as the main objective of this study was to determine the trend of practice among the respondents. However, data on the reason why the respondents did not practise HBPM are equally important to explore obstacles and consequently assist and promote the practise of HBPM among patients accordingly. We strongly suggest that future researchers incorporate this significant objective into their studies.

### Study implications

To the best of our knowledge, this study provides a comprehensive tool for assessing the knowledge, attitude and practice of HBPM among patients with hypertension by adopting the content items from previous studies. The tool can be applied to any layperson other than patients with hypertension and can be used anywhere around the world. The tool can also help doctors in assessing their patients before recommending and promoting HBPM to them.

We offer several recommendations, including strategies that empower the implementation and practice of HBPM on a larger scale and focus on specific target groups that could benefit from HBPM practice. Information about HBPM in terms of implementation, techniques and interpretation has been included in the Malaysian Clinical Practice Guideline on Hypertension 2018 for the reference of healthcare professionals. However, a manual on HBPM that is easier to understand should also be developed for patients with hypertension. This approach should be initiated through collaboration between the Health Education and Communication Centre and NCD Sector in the Ministry of Health (MOH) Malaysia.

The next recommendation is to include the HBPM practice questionnaire in the Malaysian National Health and Morbidity Survey (NHMS). The NHMS is conducted regularly every 5 years in Malaysia since 1986 by the Institute for Public Health, MOH Malaysia and covers a wide range of NCDs including hypertension. Additionally, we suggest the extension of this study to a wider variety of populations in all states of Malaysia to determine the national prevalence of HBPM practice among patients with hypertension. This information is crucial for relevant stakeholders, including the MOH Malaysia, in making decisions pertaining to HBPM practice.

Engagement with other stakeholders, such as non-government organisations, institutions and politicians, could help in gaining financial aid to purchase HBPM devices for patients in clinics as well as increase community awareness about HBPM. Frequent and scheduled training for patients organised by the MOH Malaysia or other stakeholders as stated above in different platforms could benefit them in terms of practising correct HBPM.

Finally, the initiative of promoting HBPM practice could be expanded to groups other than patients with DM, such as those who visit quit smoking clinics and tuberculosis clinics as well as the community, through MyChampion programmes (community health agents) under the MOH Malaysia such as Communication for Behavioural Impact (COMBI), *Komuniti Sihat Perkasa Negara* (KOSPEN) and Health Clinic Advisory Panel *(Panel Penasihat Klinik Kesihatan).*

## Conclusion

In this study, the knowledge and practice of HBPM among patients with hypertension in Putrajaya, Malaysia, were found to be excellent.

The important factors associated with HBPM practice included having medical insurance, having DM, being a non-smoker, staying with household members and engaging in physical activities.

## Data sharing statement

All information obtained in this study will be kept confidential and will not be made publicly available to the extent permitted by law.

**How does this paper make a difference in general practice?**

- The items on knowledge, attitude and practice of home blood pressure monitoring (HBPM) in the validated questionnaire can be used to assess the readiness of patients with hypertension to apply HBPM as a non-pharmacological intervention to control BE
- Doctors can advise patients with hypertension on the correct practice of HBPM based on the results of the questionnaire.
- The findings of this study can be used as a reference to replicate a larger-scale study and assist in policy-making in the future.

## Appendix 1

Items on the knowledge of HBPM.

**Table:**

No.	Item	Answer
1.	Exercise should be avoided at least 30 minutes before taking blood pressure measurement.	No ***Yes***
2.	Smoking should be avoided at least 30 minutes before taking blood pressure measurement.	No ***Yes***
3.	Consuming food/drinks containing caffeine should be avoided at least 30 minutes before taking blood pressure measurement.	No ***Yes***
4.	How long should you rest and relax without any distractions?	Less than 5 minutes ***More than 5 minutes***
5.	What is the dressing requirement during measurement?	***Wear loose-fitting clothes and expose the upper arm for wrapping the cuff around it.*** Wear tight-fitting clothes and there is no need to expose the upper arm to wrap the cuff around it.
6.	What are the appropriate characteristics of tables and chairs used for blood pressure measurement?	A stable table and chair of height below the heart level with an armrest ***A stable table and chair of appropriate height (at the heart level) with an armrest***
7.	Blood pressure measurement devices should be calibrated.	No ***Yes***
8.	Where should you put the cuff?	The lower border of the cuff should be placed exactly at the pit of the elbow. ***The lower border of the cuff should be placed 2 cm above the pit of the elbow, which is approximately two fingerbreadths.***
9.	Where should be the location of the cuff tubing?	***The cuff tubing is placed at the side of your arm.*** The cuff tubing is placed at the centre of your arm facing the front.
10.	What is the appropriate tightness of the cuff?	You should not be able to just slip two fingertips beneath the cuff, near its edge at the top end. ***You should be able to slip two fingertips beneath the cuff, near its edge at the top end.***
11.	How do you measure your blood pressure?	***Sit down, put on the cuff, press the button, blood pressure reading on the screen monitor, release the cuff.*** Put on the cuff, sit down, press the button, release the cuff, blood pressure reading on the screen monitor.
12.	When can you repeat the measurement of your blood pressure?	Repeat measurement immediately (less than 1 minute) after complete release of the cuff. ***Repeat measurement at least 1 minute after complete release of the cuff.***
13.	Can you talk/move during blood pressure measurement?	Yes ***No***
14.	How do you write your blood pressure reading?	***Systolic reading/diastolic reading (mmHg), e.g. 140/80 mmHg*** Diastolic reading/systolic reading (mmHg), e.g. 80/140 mmHg
15.	What is the cut-off point for blood pressure control at home?	***Should be less than 135/85 mmHg*** Should be less than 140/90 mmHg
16.	What is the cut-off point for blood pressure control at the clinic?	Should be less than 135/85 mmHg Should be less than 140/90 mmHg

Correct answer is indicated by *italic* and **bold** fonts.

Items on the attitude towards HBPM.

**Table:**

No.	Item	Scale				
1.	Home blood pressure monitoring is important in the management of hypertension (***positive statement***).	1	2	3	4	5
2.	Home blood pressure monitoring is beneficial to reduce blood pressure (***positive statement***).	1	2	3	4	5
3.	Home blood pressure monitoring is not easy to perform (***negative statement***).	1	2	3	4	5
4.	Home blood pressure monitoring can be done by anyone (***positive statement***).	1	2	3	4	5
5.	Home blood pressure monitoring is costly (***negative statement***).	1	2	3	4	5
6.	The blood pressure reading from home blood pressure monitoring is not accurate (***negative statement***).	1	2	3	4	5
7.	The information or education leaflet given to me is really helpful for using home blood pressure monitoring (***positive statement***).	1	2	3	4	5
8.	I would like to recommend others to practise home blood pressure monitoring (***positive statement***).	1	2	3	4	5
9.	I am satisfied only with the blood pressure measurement in the clinic (***negative statement***).	1	2	3	4	5

Item scale: 1 - Strongly agree, 2 - Agree, 3 - Neutral, 4 - Disagree, 5 - Strongly disagree

## References

[1] Mills KT, Bundy JD, Kelly TN (2016). Global disparities of hypertension prevalence and control: a systematic analysis of population-based studies from 90 countries.. Circulation..

[2] Hamrahian SM, Falkner B (2017). Hypertension in chronic kidney disease. Hypertension: from basic research to clinical practice, Volume 2. Adv Exp Med Biol.. Springer Publications..

[3] World Health Organization. (2021). Fact Sheet - Hypertension..

[4] Hodgkinson J, Mant J, Martin U (2011). Relative effectiveness of clinic and home blood pressure monitoring compared with ambulatory blood pressure monitoring in diagnosis of hypertension: systematic review.. BMJ..

[5] Mills KT, Stefanescu A, He J (2020). The global epidemiology of hypertension.. Nat Rev Nephrol..

[6] Ministry of Health Malaysia. (2011). National Health & Morbidity Survey 2011. Volume II: Non-Communicable Diseases..

[7] Ministry of Health Malaysia. (2015). National Health & Morbidity Survey 2015. Volume II: Non-Communicable Diseases, Risk Factors & Other Health Problems..

[8] Ministry of Health Malaysia. (2020). National Health & Morbidity Survey 2019. Non-communicable diseases, healthcare demand, and health literacy: Key Findings..

[9] Ministry of Health Malaysia. (2023). National Health & Morbidity Survey 2023. Non-communicable diseases and healthcare demand: Key Findings..

[10] Chia YC, Buranakitjaroen P, Chen CH (2017). Current status of home blood pressure monitoring in Asia: statement from the HOPE Asia Network.. J Clin Hypertens..

[11] Shimbo D, Artinian NT, Basile JN (2020). Selfmeasured blood pressure monitoring at home: a joint policy statement from the American Heart Association and American Medical Association.. Circulation..

[12] Stergiou GS, Bliziotis IA (2011). Home blood pressure monitoring in the diagnosis and treatment of hypertension: a systematic review.. Am J Hypertens..

[13] Bobrie G, Chatellier G, Genes N (2004). Cardiovascular prognosis of masked hypertension detected by blood pressure selfmeasurement in elderly treated hypertensive patients.. JAMA..

[14] Bonafini S, Fava C (2015). Home blood pressure measurements: advantages and disadvantages compared to office and ambulatory monitoring.. Blood Press..

[15] Ayala C, Tong X, Keenan NL (2012). Regular use of a home blood pressure monitor by hypertensive adults—HealthStyles, 2005 and 2008.. J Clin Hypertens..

[16] Wake AD, Bekele DM, Tuji TS (2020). Knowledge and attitude of self-monitoring of blood pressure among adult hypertensive patients on follow-up at selected public hospitals in Arsi Zone, Oromia Regional State, Ethiopia: A cross-sectional study.. Integr Blood Press Control..

[17] Cuspidi C, Meani S, Lonati L (2005). Prevalence of home blood pressure measurement among selected hypertensive patients: results of a multicenter survey from six hospital outpatient hypertension clinics in Italy.. Blood Press..

[18] Chudek A, Owczarek AJ, Ficek J, Almgren-Rachtan A, Chudek J (2020). Lower utilization of home blood pressure monitoring in younger, poorly educated hypertensive males-real-life data.. Blood Press..

[19] Seidlerova J, Filipovsky J, Wohlfahrt P, Mayer Jr O, Cifkova R (20l4). Availability and use of home blood pressure measurement in the Czech general population.. Cor Vasa..

[20] Lau E, Kaczorowski J, Karwalajtys T, Dolovich L, Levine M, Chambers L (2006). Blood pressure awareness and self-monitoring practices among primary care elderly patients.. Can Pharm J..

[21] Viera AJ, Cohen LW Mitchell CM, Sloane PD (2008). Use of home blood pressure monitoring by hypertensive patients in primary care: survey of a practice-based research network cohort.. J Clin Hypertens..

[22] Springer MV, Malani P, Solway E (2022). Prevalence and frequency of self-measured blood pressure monitoring in US adults aged 50-80 years.. JAMA Netw Open..

[23] Tan N, Khin L, Pagi R (2005). Home blood-pressure monitoring among hypertensive patients in an Asian population.. J Hum Hypertens..

[24] Lydakis H, Tzinas J, Athousakis E, Mihou E (2006). Use of home blood pressure measurement by hypertensive patients in southern Greece.. J Hum Hypertens..

[25] Department of Statistic Malaysia. (2020). Household Income & Basic Amenities Survey Report 2019..

[26] Fu SN, Dao MC, Wong CK, Cheung BM (2022). Knowledge and practice of home blood pressure monitoring 6 months after the risk and assessment management programme: does health literacy matter?. Postgrad Med J..

[27] Fu SN, Dao MC, Wong CKH, Cheung BMY (2020). The association of health literacy with high-quality home blood pressure monitoring for hypertensive patients in outpatient settings.. In't J Hypertens..

[28] Harpe SE (2015). How to analyze Likert and other rating scale data.. Curr Pharm Teach Learn..

[29] World Health Organization. (2020). WHO Technical Specifications for Automated NonInvasive Blood Pressure Measuring Devices With Cuff..

[30] United States Centers for Disease Control. (1988). Body Measurements (Anthropometry)..

[31] Ambakederemo T, Ebuenyi I, Jumbo J (2014). Knowledge and attitude to self-monitoring of blood pressure in a cardiology clinic in Nigeria.. IOSR J Dent Med Sci..

[32] Alotaibi A, Alotaibi A, Alsomali A (2023). Evaluation of knowledge and practices of hypertensive patients regarding home blood pressure monitoring.. Fam Med Prim Care Rev..

[33] Breaux-Shropshire TL, Brown KC, Pryor ER, Maples EH (2012). Prevalence of blood pressure selfmonitoring, medication adherence, self-efficacy, stage of change, and blood pressure control among municipal workers with hypertension.. Workplace Health Saf..

[34] Shafie A, Hassali M (2013). Willingness to pay for voluntary community-based health insurance: findings from an exploratory study in the state of Penang, Malaysia.. Soc Sci Med..

[35] Gregory S (2005). Living with chronic illness in the family setting.. Sociol Health Illn..

[36] Baig AA, Benitez A, Quinn MT, Burnet DL (2015). Family interventions to improve diabetes outcomes for adults.. Ann N Y Acad Sci..

[37] Adu MD, Malabu UH, Malau-Aduli AE, Malau-Aduli BS (2019). Enablers and barriers to effective diabetes self-management: a multi-national investigation.. PLoS One..

[38] Zhang F, Or PP, Chung JW (2021). How different health literacy dimensions influences health and well-being among men and women: the mediating role of health behaviours.. Health Expect..

[39] Islam FMA, Islam MA, Hosen MA (2023). Associations of physical activity levels, and attitudes towards physical activity with blood pressure among adults with high blood pressure in Bangladesh.. PLoS One..

